# Episodic memory development: Bridging animal and human research

**DOI:** 10.1016/j.neuron.2024.01.020

**Published:** 2024-04-03

**Authors:** Juraj Bevandić, Loïc J. Chareyron, Jocelyne Bachevalier, Francesca Cacucci, Lisa Genzel, Nora S. Newcombe, Faraneh Vargha-Khadem, H. Freyja Ólafsdóttir

**Affiliations:** 1 Donders Institute for Brain, Cognition and Behaviour, Radboud University, Nijmegen, the Netherlands; 2 Cognitive Neuroscience and Neuropsychiatry, Developmental Neurosciences, University College London Great Ormond Street Institute of Child Health, London, UK; 3 Laboratory of Brain and Cognitive Development, Institute of Psychology, University of Lausanne, Lausanne, Switzerland; 4 Division of Developmental and Cognitive Neuroscience, Emory National Primate Research Center, Department of Psychology, Emory University, Atlanta, GA, USA; 5 Department of Neuroscience, Physiology and Pharmacology, University College London, London, UK; 6 Department of Psychology, Temple University, Philadelphia, PA, USA

**Keywords:** episodic memory, development, hippocampus, human, rodent, non-human primate, W-W-W memory

## Abstract

Human episodic memory is not functionally evident until about 2 years of age and continues to develop into the school years. Behavioral studies have elucidated this developmental timeline and its constituent processes. In tandem, lesion and neurophysiological studies in non-human primates and rodents have identified key neural substrates and circuit mechanisms that may underlie episodic memory development. Despite this progress, collaborative efforts between psychologists and neuroscientists remain limited, hindering progress. Here, we seek to bridge human and non-human episodic memory development research by offering a comparative review of studies using humans, non-human primates, and rodents. We highlight critical theoretical and methodological issues that limit cross-fertilization and propose a common research framework, adaptable to different species, that may facilitate cross-species research endeavors.

## Teaser

Bevandić et al. review human, non-human primate, and rodent studies on the ontogeny of episodic and episodic-like memory. They identify theoretical and methodological obstacles that currently limit cross-species insights and propose a common research framework to facilitate comparative studies.

## Introduction

“Episodic memory” is the ability to anchor memories for events in our lives to their spatiotemporal context and the ability to recall these at later times^1^ (“what-where-when” [W-W-W] memory^2^). It is central to our sense of personal identity and supports adaptive everyday decision-making and planning. Although the developing infant is a prodigious learner, able to rapidly assimilate world knowledge and acquire language proficiently, the ability to encode and recall detailed episodic memories develops relatively late.^3^^,^^4^ Specifically, adults cannot recall episodic memories from the first ∼2 years of life,^5^^,^^6^^,^^7^ although the ability to encode simple items develops early in infancy, along with the ability to learn facts about the world.^3^^,^^8^^,^^9^^,^^10^ By ∼2 years of age, children can form elemental W-W-W memories^5^^,^^6^^,^^7^—such as knowing the spatial location of a reward.^5^^,^^7^ The ability to encode detailed W-W-W memories—where multiple items need to be bound to their spatiotemporal context—and to retain these over extended periods of time continues to mature until the school years and beyond.^4^^,^^11^^,^^12^^,^^13^^,^^14^ The inability of adults to retrieve episodic memories from their early lives has been termed infantile amnesia.^15^ However, we refrain from using this term as it carries a clinical connotation, which is odd in the context of healthy cognitive development.

Lesion and stereological studies carried out on non-human primates (NHPs) and rodents have highlighted that the protracted development of episodic memory likely depends, at least in part, on the hippocampus,^16^^,^^17^^,^^18^ a brain structure important for such memory in adults.^19^ Selective lesions of the hippocampus in developing monkeys, for example, prevent the maturation of forms of W-W-W memory.^20^^,^^21^ Further, in recent years, significant advances have been made to our understanding of the neurophysiological basis of episodic memory ontogeny, as neural recording and perturbation tools have started to be applied to the living, developing rodent brain. This research has, for example, elucidated the ontogeny of functional neuronal representations,^22^^,^^23^^,^^24^ network mechanisms,^25^^,^^26^ and oscillations^27^ thought to contribute to mature episodic memory. Thus, the field is at a critical stage where we are starting to gain unprecedented insight into the cognitive-neurobiological building blocks of episodic memory development.

Despite this progress, comparative efforts between neuroscientists and psychologists have remained limited. Carefully designed tasks set up to measure specific facets of episodic memory in developing children are rarely used in non-human animal studies, and some tasks are not translatable across species. In addition, debates remain regarding whether W-W-W memory truly captures the concept of episodic memory, in part because the term has undergone some revision since its first description. Tulving^28^^,^^29^ proposed that in addition to being a W-W-W memory, a “true” episodic memory is retrieved via conscious recall (“autonoesis,” see Box 1 for glossary). However, as “autonoetic recall” can only be assessed through introspection and linguistic report, including this criterion has presented significant challenges for studying the cognitive and physiological basis of episodic memory ontogenesis in non-human animals that lack language, and even in children with limited verbal abilities.

Box 1 **Glossary**

**Episodic memory**: memory for personal events that can be consciously recalled. Episodic memory includes information about what happened where and when (W-W-W).

**What-where-when (W-W-W) memory/episodic-like memory**: what-where-when memory where conscious recall cannot be ascertained. Term is commonly used when investigating episodic memory in non-human animals and pre-verbal infants.

**Semantic memory**: memory for facts and world knowledge (e.g., knowing Paris is the capital of France vs. remembering your last trip to Paris). Unlike episodic retrieval, retrieval of semantic memories is usually not associated with a felt sense of traveling back to where/when encoding took place.

**Encoding**: the ability to form a memory trace.

**Recognition**: the ability to recognize previously encoded stimuli as old without the need to retrieve the encoded memory trace. Recognition is usually thought to be supported by familiarity processes and can be tested via VPC, OR, and multiple-choice paradigms, although recognition tests may evoke recall processes when deep processing is encouraged during study.

**Recall**: the ability to retrieve an encoded memory from memory storage. Recall is traditionally tested via strategic recall (i.e., where subjects are simply asked to freely recall studied stimuli, such as a list of words) but can also be tested via spontaneous recall—where subjects are provided with a distinctive cue of the encoded memory.

**Autonoesis**: conscious recall experienced and reported from a first-person perspective. During autonoetic recall the person experiences traveling back in time to retrieve a stored memory.

**Pattern separation**: the process by which overlapping or similar memories are supported by non-overlapping neuronal representations.

**Allocentric memory**: spatial memory encoded in an environment-centric reference frame as opposed to an egocentric reference frame (e.g. the train station is located north of the town hall vs. the train station is on my right).

Our aim here is to bridge the complementary work of researchers investigating different mammalian species by offering a comparative review through the lens of the original W-W-W memory framework.^2^ Specifically, we will review research carried out in humans, NHPs, and rodents to describe key cognitive and neurobiological developmental milestones of W-W-W memory. We will highlight theoretical and methodological caveats that currently limit cross-species translational impact and identify critical knowledge gaps that remain. Although W-W-W memory may be a mere proxy for human episodic memory capability—sometimes termed episodic-like memory^30^—we believe it represents a valuable tool for studying the ontogeny of this core cognitive capability in comparative research settings.

We structure the review around specific facets of W-W-W memory (i.e., object [“what”], spatial [“what-where”], spatiotemporal [W-W-W] memory) and discuss the development of encoding and retention/retrieval separately. We will use the terms W-W-W and episodic-like memory interchangeably to refer to memory that fulfills Tulving’s original definition of episodic memory^2^ but where autonoesis cannot be ascertained. Our treatment builds on prior reviews (e.g., Josselyn and Frankland,^31^ Donato et al.,^32^ Alberini and Travaglia,^33^ Donato et al.,^34^ Keresztes et al.,^35^ and Cossart and Khazipov^36^) by drawing links between species from two different mammalian groups, discussing cross-species findings explicitly side-by-side and reviewing the literature for the different components of W-W-W memory individually. We hope that adopting this methodical and comparative approach will offer novel insights into the ontogeny of episodic(-like) memory, may stimulate greater cross-fertilization, and inspire the establishment of comparative research partnerships.

## Development of episodic-like encoding

We begin by summarizing research on the development of episodic-like memory encoding, i.e., studies where memory testing occurs shortly (<∼2–10 min) after stimuli sampling.

### The development of object (what) encoding

**Figure 1:**
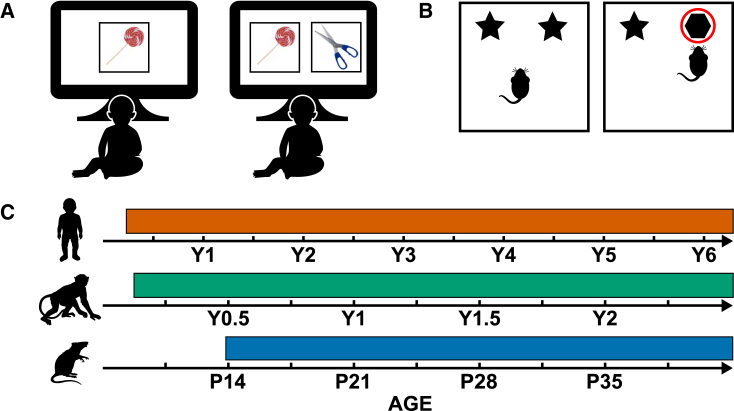
Development of what (object) encoding (A and B) Schematic diagram of a visual-paired comparison paradigm (A), and (B) an object recognition task. (C) Developmental timeline of human, non-human primate, and rodent what encoding.

A key building block for W-W-W associative memory is the ability to encode an object’s identity, i.e., the what in W-W-W memory. A wealth of studies, carried out in various mammalian species, have shown that the ability for what encoding matures first. To study object encoding, developmental researchers often rely on the visual-paired comparison (VPC) task (Figure 1A). The VPC task taps into a developing organism’s natural tendency to look more at novel visual stimuli over familiar stimuli. VPC studies have made an important contribution to memory development research, as they can be carried out in pre-verbal infants (as well as older children and even adults with some adaptation) and are amenable to cross-species testing. In humans, novelty preferences on the VPC task have been documented in infants and even neonates.^37^^,^^38^^,^^39^

Similarly, in NHPs, what encoding has been found to develop in the early post-natal period. By 1.5 months mature object encoding has been observed.^40^ When comparing NHP age with human age, one NHP month is thought to approximate 4 months in human life, until 2 years of age at least.^41^ Thus, the developmental trajectory of what encoding is comparable in humans and NHPs.

To study the development of object encoding in rodents, researchers typically rely on object recognition (OR) tasks. In OR tasks, rodents explore an environmental arena containing two identical objects. After a short delay, the rodent is placed back into the same arena, which now contains one of the original objects and one novel object (Figure 1B). If rodents have encoded the object successfully, they should preferentially explore the novel object over the familiar object. Studies have shown that mature OR encoding can be observed reliably from post-natal day 14 (P14).^42^^,^^43^^,^^44^ Comparing rodent age—counted in days or weeks—with human age is difficult. A 1-week-old rodent is often considered the rough equivalent of a newborn human, on the basis of brain oscillatory patterns.^36^^,^^45^ In terms of W-W-W memory development, multiple studies suggest that the initial inflection points occur ∼3 weeks of age (e.g., Ramsaran et al.,^24^ Douglas et al.,^46^ Campbell and Spear,^47^ and Travaglia et al.^48^). Consequently, a 3-week-old rodent may be comparable to a 2-year-old child in terms of memory maturation. Thus, the ontogeny of what encoding precedes the development of associative W-W-W encoding, a similar pattern to that observed in human and NHP development. Figure 1C shows a summary developmental timeline of what encoding.

### The development of what-where encoding

The ability to associate an object, or multiple objects, to a spatial context and/or a spatial location—i.e., what-where encoding—is a key component of W-W-W memory. For simplicity, we define the term “context” broadly, as is common in the contemporary literature, to refer to the background on which an object is displayed, the environment in which an object is located and/or the specific features of an object’s environment. The phrase “spatial position” refers to the “allocentric” (environment-centric) spatial location of a stimulus within an environment rather than its egocentric location. We review the literature that has charted the ontogeny of these two related forms of what-where encoding.

#### Contextual what-where encoding

**Figure 2:**
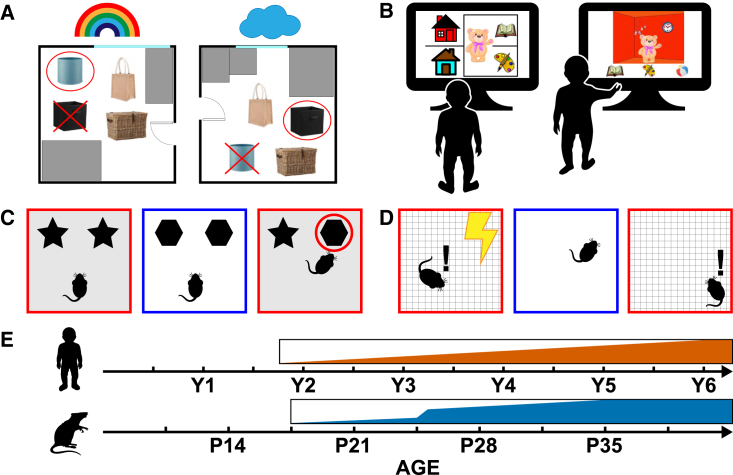
Development of contextual what-where encoding (A–D) (A) Schematic of a contextual memory task used in Newcombe et al.,^54^ (B) multi-item contextual memory task from Ngo et al.,^12^ (C) object-context recognition, and (D) contextual fear conditioning. (E) Developmental timeline of human and rodent contextual encoding.

Some studies have found contextual modulation of visual preferences on VPC tasks in infants as young as 6 months of age,^49^ possibly suggesting that contextual what-where encoding may emerge in the neonatal period. Similarly, Rovee-Collier and colleagues have shown that contextual features (crib bumper designs) influence the ability of infants to display a learned action association (a kick leading to movement in an overhead mobile).^50^ However, studies that carefully control for the type of processing required for novelty detection suggest that what may appear as contextual what-where encoding early in infancy may rather reflect encoding of an inflexible, compound representation.^51^^,^^52^^,^^53^ Specifically, Robinson and Pascalis^51^ showed that 12-month-old infants displayed similar novelty preferences for an image containing an object observed before but presented on a novel background and a completely novel object-background image. However, 18- to 24-month-old toddlers preferentially looked at the novel object-background image,^51^ suggesting that this age marks the onset of flexible contextual what-where encoding. This pattern agrees with findings from Newcombe and colleagues^54^ who had children learn the location of a distinctive toy in two different rooms. Newcombe et al. found that 21- to 26-month-old children, if presented with a cue, were able to identify the container that had the toy for a given context, whereas 15- to 21-month-old children tended to search in both of the two containers that were rewarded in either context (Figure 2A). The authors also noted significant improvement in the ability of older children (3–5 years) to discriminate between the two contexts, with near ceiling performance on this simple task observed at 5 years of age. Thus, what-where encoding displays an inflection point at ∼2 years of age in humans but continues to mature until at least age 5.

Although less is known about the development of contextual what-where encoding in NHPs, much attention has been paid to its development in rodents. To study contextual encoding, rodent researchers have traditionally used contextual fear conditioning (CFC) paradigms. In these tasks rodents are placed into a neutral environment where they experience a series of (mild) electric foot shocks. To determine whether the rodent has learned the contextual association, the experimenter places the pup back into the environment after some delay and assesses whether the pup freezes upon re-exposure (Figure 2D). A number of studies have found that contextual encoding measured in this way is already apparent in the pre-weaning period,^55^^,^^56^^,^^57^ with the earliest onset reported at P13.^56^ However, recent evidence suggests that these early emerging contextual memories may lack specificity, and that precise, adult-like contextual encoding does not emerge until the fourth post-natal week.^24^

Similar findings have been observed in object-context recognition (OCR) studies. In these studies, rodents are presented with a pair of identical objects in one environment and another object pair in a second environment. At testing, the animals are presented with one contextually familiar object (explored in that environment before) and one contextually novel object (explored in a different context). Preferential exploration of the contextually novel object is interpreted as a measure of successful object-context encoding (Figure 2C). Using this procedure, Ramsaran and colleagues^58^^,^^59^ found evidence for object-context encoding at P17.^58^ However, these results are at odds with Asiminas et al., who only found novelty preferences on the OCR task in pups in the fifth post-natal week.^60^ Although the reason for these discrepant results remains to be ascertained, there were notable methodological differences between the two studies. For Ramsaran et al. context meant a completely different environment, located in a different room, with different set of local and distal cues, whereas Asiminas et al. altered only the floor and wall texture/color. Indeed, Ramsaran and colleagues showed that the ability to encode object-context associations when context is only defined in terms of the location of distal cues displays a significantly later inflection point (P26).^58^

Thus, akin to CFC studies, the OCR studies suggest that the ability to form precise contextual associations emerges only in the post-weaning period, likely during fourth and fifth post-natal weeks. Overall, it seems that contextual what-where encoding develops relatively late in mammals and that the initial inflection points observed in individual species occur at comparable developmental stages. Figure 2E shows a summary developmental timeline of contextual what-where encoding.

#### Spatial what-where encoding

Early studies using VPC paradigms suggested that the encoding of spatial location of an object on a visual display was already mature in infancy.^61^ Similar observations have been noted in spatial location VPC studies carried out in NHPs.^62^ However, the robustness of this early emerging what-where spatial encoding has been brought into question. It is unclear whether novelty preferences observed in human and NHP infancy reflect what-where processing within an allocentric reference frame, or whether they could be accounted for by simpler egocentric or fused (treating the object-background stimulus as a unified scene) encoding strategies,^62^^,^^63^ as has been suggested for contextual what-where encoding.^51^^,^^52^

**Figure 3:**
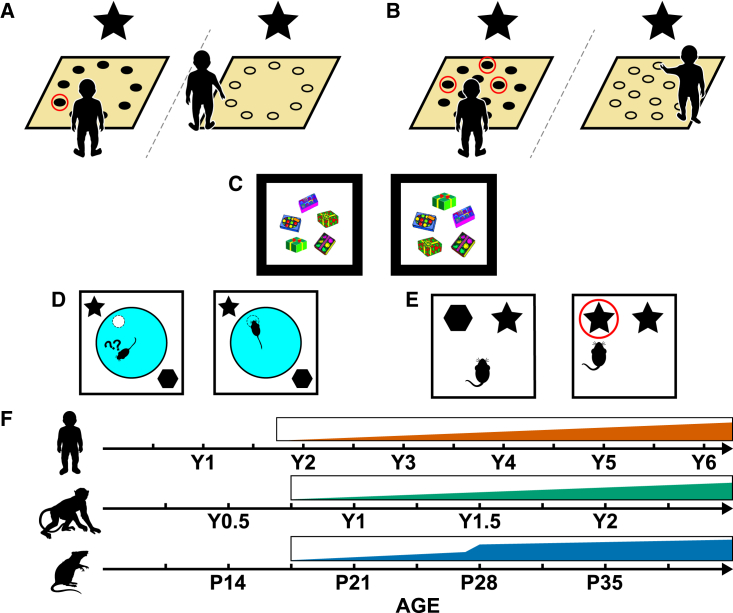
Development of spatial what-where encoding (A–E) (A) Schematic of single-location place memory task,^5^ (B) three-location place memory task,^7^ (C) object-in-place VPC,^62^ (D) the watermaze, and (E) an object-place recognition paradigm. (F) Developmental timeline of human, NHP, and rodent spatial what-where encoding.

Studies that have specifically investigated the ontogeny of allocentric spatial encoding, suggest a later inflection point. At ∼2 years of age children start being able to use landmarks to guide their search for a reward in an open arena^5^^,^^7^ (Figure 3A), a hallmark of allocentric spatial encoding. Similarly, by 9 months of age, NHPs show the ability to use landmarks to guide their search for food in an open arena.^64^ Thus, both humans and NHPs display a relatively late inflection point for allocentric, spatial what-where encoding and this inflection point mirrors that observed for contextual encoding.

Comparable observations have been reported in rodent development. The ability to recognize a displaced object in a familiar environment (object-location recognition [OLR] task, i.e., location of object within environment changes between encoding and testing) has been observed during the third post-natal week.^44^^,^^48^ However, a version of this task—so-called object-place recognition (OPR, Figure 3E)—which requires animals to form a more specific object-place association—location in which an object appears is not novel, but the conjunction of a particular object in a particular place is—displays a later inflection, with novelty preferences first reported at 4 weeks of age.^65^ Similarly, studies assessing the development of the ability to locate a hidden escape platform in a murky pool using landmark cues (watermaze^66^; Figure 3D) have shown that this capability only becomes adult-like at ∼4 weeks of age.^67^^,^^68^^,^^69^ Some studies have observed an earlier emergence of allocentric memory in the watermaze.^70^ However, when the strategies used by developing rodents to encode the location of the escape platform are methodically assessed—by changing the spatial relationship between the pool and distal, landmark cues—it seems the early emerging what-where encoding in the watermaze is likely supported via the encoding of local cues and/or the implementation of a directional strategy rather than a landmark guided, allocentric strategy.^67^^,^^69^

Thus, akin to human and NHP development, spatial what-where encoding develops relatively late in rodents (see Figure 3F for a summary timeline of spatial what-where encoding development). However, as important inflection points seem to only occur at ∼4 weeks of age in rodents, this may suggest that this form of W-W-W encoding matures at a relatively later age in rodents compared with humans and NHPs. Alternatively, the age of this inflection point could be influenced by the late development of the rodent visual system, which does not become adult-like until 6–7 weeks of age.^71^ Indeed, visual acuity is known to affect performance on the watermaze in adult rats.^72^ Perhaps future studies can investigate the development of allocentric encoding using cues that draw on the early developing senses, such as audition or olfaction, such that the ontogeny of allocentric encoding can be more readily compared in different mammalian species.

#### Multi-item what-where encoding

The ability to bind multiple objects to a given contextual and spatial location continues to grow in complexity after single item what-where encoding has emerged. Ngo and colleagues had 4- and 6-year-old children watch cartoons in which different houses contained pairs of items that varied by context, e.g., “a bear in the red house is holding a painting palette but in the blue house it is holding a book.” Following the encoding session, the children were shown one of the two contexts containing only the overlapping item (e.g., bear in the red house). The children’s task was to correctly identify which item the bear had been paired with in that context (painting palette). Importantly, among the items the children could choose from was a lure (book) as well as foils (e.g., ball) (Figure 2B). Ngo and colleagues found that 6-year-old children were significantly better at discriminating between target and lure items compared with 4-year-olds. Indeed, 4-year-old children’s ability to reject lures did not differ from chance, while their memory for individual items was relatively intact.^12^ In the spatial domain, Ribordy and colleagues showed that between the ages of 3 and 5, children start being able to encode the locations of multiple rewards in an open arena within an allocentric reference frame^7^ (Figure 3B), similar to results obtained by Overman and colleagues.^73^ Thus, the ability to form complex what-where memories—where multiple items need to be bound to their context or allocentric spatial location—undergoes significant development between the ages of 4 and 6.

NHP primate studies have also observed a late inflection point for multi-item what-where binding. Blue et al. found novelty preference on the object-in-place VPC task—where monkeys need to encode the spatial location of multiple objects in an array—only emerges near the end of the 2nd post-natal year (J. Bachevalier, unpublished data)^62^ (Figure 3C). These studies suggest that multi-item binding emerges relatively late in NHP development as it does in human development. We know of no rodent study that has charted the ontogeny of multi-item what-where encoding.

The improvements in what-where contextual and spatial encoding observed may reflect development in the resolution of the memory representation. Ngo and colleagues showed that accurate discrimination of two contextually similar memories (occurring in two similar houses) did not differ between 4- and 6-year-olds, but their performance was lower compared with adults.^74^ Similarly, Lambert and colleagues showed that the ability to discriminate between nearby spatial locations and temporally close memories improved between the ages of 3.5 and 7.^13^

In sum, a hallmark of episodic-like memory encoding development may be the emergence of the ability to form complex and specific what-where memories. This suggestion agrees with contemporary theories of episodic memory development that posit that one of the key changes to cognition early in life is the emergence and refinement of pattern separation,^12^^,^^35^^,^^75^ which supports orthogonal encoding of individual but overlapping memories.

### The development of what-when and W-W-W encoding

**Figure 4:**
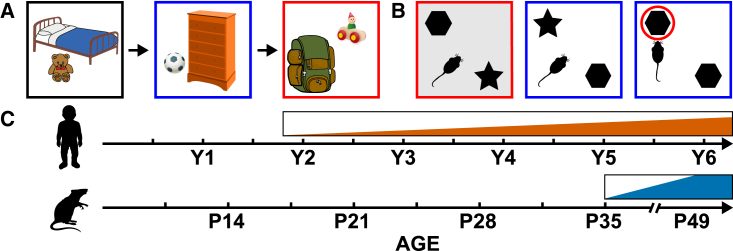
Development of what-where-when encoding (A and B) (A) Schematic of the what-where-when memory task used in Hayne and Imuta,^76^ and (B) object-place-context recognition paradigm. (C) Developmental timeline of human and rodent what-where-when encoding.

The emergence of temporal binding in W-W-W memory as well as the full W-W-W encoding triad has been studied less systematically. When studying the encoding of time in W-W-W memory, researchers often assess the ability of children to encode the order or sequence in which events occur. One test of temporal encoding is the “hide and seek” test used by Hayne and Imuta.^76^ In their study, 3- and 4-year-old children observed an experimenter hide three distinct toys in different rooms in their house. At testing, 5 min later, the experimenter asked the children to tell them in what order they had entered the different rooms (i.e., “what-when” memory) (Figure 4A). The authors found 3-year-olds performed significantly worse than 4-year-olds, while both age groups were able to accurately remember where the toys had been hidden (what-where memory). This pattern suggests what-when encoding may lag the development of what-where memory. Similarly, Mastrogiuseppe and colleagues^77^ found that what-when memory (order in which three objects were hidden) develops particularly late (6–8 years of age), lagging behind where-when memory (knowing which locations contained the same hidden object and the order in which objects were placed in those locations). Its emergence coincided with the development of W-W-W memory (knowing the order in which three distinct objects were placed in three different locations).^77^ However, testing took place after an interference task, making it hard to determine if the late emergence of what-when and W-W-W memory reflects the inability to encode such associations or the effect of memory interference early in life.

In contrast, early studies using deferred imitation^78^ to study the development of temporal encoding—where infants/toddlers observe an adult perform a sequence of actions and are then tested for their memory of the action sequence—have shown a relatively early inflection point. Successful encoding of arbitrary action sequences has been observed near the end of the second year.^79^ VPC studies assessing memory for temporal order—where children watch familiar movie clips whose temporal sequence has been scrambled—have observed an even earlier inflection point. Sonne and colleagues found evidence for what-when temporal encoding in infancy (down to 6 months of age).^80^^,^^81^ Although the reasons for these discrepant findings remain to be ascertained, Sonne et al. showed children clips that displayed scenes whose normal temporal order is dictated by physics (e.g., jumping up and down). Thus, novelty preferences in the scrambled clips could have been influenced by the fact that these clips violated the infants’ expectations of physical regularities. More in line with Mastrogiuseppe et al., Benear and colleagues showed that memory for the temporal order of complex events develops significantly between the ages of 4 and 7.^82^

The ontogeny of what-when and W-W-W memory in non-human animals has received relatively little research attention. This likely reflects the difficulty in assessing temporal encoding in non-human animals. However, researchers have used the so-called object-place-context recognition (OPCR) memory test as a proxy to study such W-W-W encoding in rodents. In the OPCR test, rodents not only have to learn to associate an object with a spatial location, but also a particular context (see Figure 4B). Context in these paradigms has been argued to represent a form of temporal encoding.^60^ Two studies have attempted to chart its ontogenetic emergence. Asiminas and colleagues found this encoding triad in the rat emerged at 7 weeks of age, while Ramsaran and colleagues observed an inflection point in 5th post-natal week.^59^ Again, methodological differences in how context was defined may explain these discrepant results—different rooms vs. changes to the color and texture of walls and floor. Further, Asiminas and colleagues tested the pups on other OR tests prior to the OPCR test, which may have caused interference. In any case, these studies provide the first indication that W-W-W, episodic-like encoding may emerge ∼5–7 weeks of age in rodents. See Figure 4C for a summary timeline of what-when and W-W-W encoding development.

It should be highlighted that paradigms for studying W-W-W memory have been developed for non-human animals, such as food caching birds (for a review see Griffiths et al.^30^) and apes.^83^ Clayton and Dickinson showed that scrub jays could remember where particular foods (peanuts or worms) were located after a single caching event and were also able to integrate information about time since caching when allowed to recover their cached items.^84^ A similar paradigm has been adapted for chimpanzees, and they show a similar capability for W-W-W encoding.^83^ We encourage the memory development research community to develop comparable paradigms that are suitable for rodents such that the development of W-W-W encoding can be more directly compared between rodents and primates.

## Development of episodic-like retention and retrieval

The previous discussion has concentrated on W-W-W memory encoding. Now we shift to discussing the development of the ability to retain new memories over time and the development of retrieval processes.

### The development of episodic-like memory retention

VPC studies, which have investigated the emergence of the what element in W-W-W memory, show that object novelty preferences can be observed in 3- to 6-month-old infants at short (24 h) delays. Further, Fagan found that 6-month-old infants already display novelty responses when a 2-week delay between presentation and testing is introduced.^37^ Using familiarity preferences as a measure of memory over longer delays, Bahrick and Pickens observed memory over a 3-month period in 3-month-old infants.^85^ In rodents, OR tasks have shown memory retention at 2- and 24-h delays at the start of the fourth post-natal week,^44^^,^^86^ although one study observed intact OR memory at P17.^87^ These data suggest that retention of simple what memories may emerge relatively early in human and rodent development. Memory retention has not been systematically tested in NHPs (but see Lavenex and Lavenex^64^).

Less is known regarding the development of retention for associative W-W-W memories. Deferred imitation studies have shown that retention of arbitrary action sequences over a 2-week period is present at the start of the third post-natal year.^79^ Benear and colleagues showed that retention over 24 h of contextual what-where memories was present in 4-year-olds but was lower than the retention observed in 6-year-olds.^88^ Further, Saragosa-Harris and colleagues found that contextual memory (remembering object-background scenes) at long delays (1 week), although present, was notably weaker in 3-year-olds compared with 5-year-olds.^11^ This pattern is in agreement with the results by Scarf et al.^89^ who studied retention using a task inspired by the so-called spoon test—a test proposed by Tulving et al.^90^ as a non-verbal test of episodic memory in children. In their study, children’s ability to encode and recall an object (key) association with a play event (treasure hunt) was tested. Scarf et al. found intact retention at 24-h and 1-week delay in 4-year-olds, while 3-year-olds only showed retention at short (up to 30 min) delays.^89^ Overall, questions remain regarding the ontogenetic timeline of W-W-W memory retention in humans, given the paucity of systematic studies.

This topic has not been studied extensively in NHPs, but there are rodent studies. For example, Travaglia and colleagues^91^ tested contextual what-where memory retention using a CFC paradigm. They found that P17 mouse pups tested 24 h after encoding showed no memory of the learned association while P24 pups did.^91^ In another study Travaglia et al. found object-location memory at a 2-h delay only at P24 and not P17.^48^ Similarly, Ramsaran and colleagues^24^^,^^92^ found robust what-where retention at 24 h in P24 pups^92^ (similar to Anderson and Riccio^93^). However, others have found evidence for contextual as well as spatial what-where memory during the third post-natal week (from P15).^56^^,^^70^^,^^87^

These contradictory findings, both in the rodent and human literature, may be explained by two factors: encoding specificity and retrieval processes. In rodent studies, the specificity of the retained spatial/contextual what-where memory is not always explicitly tested. For example, if testing contextual what-where retention using a CFC paradigm, studies do not routinely place the pups into a neutral environment just after the encoding session to ascertain that the learned association is specific to the shock environment. Although this has been done in some cases at memory testing sometime after encoding (e.g., Ramsaran et al.^24^), not assessing the specificity of the contextual association immediately after encoding makes it hard to interpret any absence of retention. That is, immaturity of contextual what-where retention could simply reflect immaturity of encoding. Somewhat similar arguments can be made for the reported early emergence of spatial what-where retention using the watermaze.^56^^,^^70^ As research has shown that encoding on this task may not be allocentric in younger pups (P20–P21),^69^ the early emergence of retention found may not reflect the emergence of allocentric spatial memory, but rather retention of egocentric spatial memories. Indeed, adult-like allocentric encoding has only been reported at P26–P27.^69^

**Figure 5:**
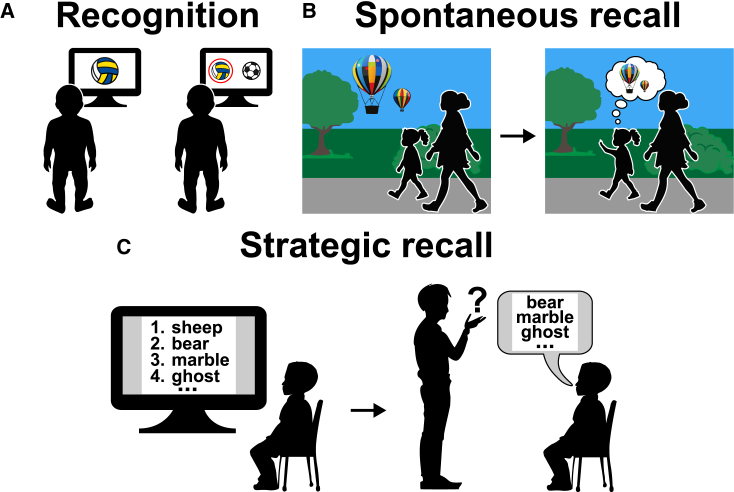
Memory retrieval processes (A–C) Schematic of a recognition (A), spontaneous recall (B), and strategic recall paradigms (C).

Concerns about encoding specificity may not readily apply to the discrepant results observed in the human literature, as appropriate encoding controls conditions are often employed.^11^^,^^88^^,^^89^ To reconcile these differences, one may need to consider retrieval processes. Studies that assess associative memory retention via novelty preferences on the VPC task are thought to only require “recognition” memory—the human/non-human animal just has to recognize that they have seen stimuli before (Figure 5A). Other studies require children to explicitly express a learned W-W-W memory, i.e., to “recall” a stored memory from long-term memory. Hence, we now turn to examine different retrieval processes.

### The development of episodic-like retrieval processes

VPC studies suggest that recognition-mediated memory retention of certain aspects of W-W-W memories mature early, perhaps being present in the first post-natal year.^37^^,^^85^ However, studies that require children to explicitly recall a memory suggest that memory retention develops notably later (between the ages of 3 and 8^11^^,^^77^^,^^89^). Adult-like (autonoetic) recall has not been studied extensively. However, autonoetic recall of personal events experienced at age 5 and tested between the ages of 6 and 11 years seems to improve with age.^14^ Older children were more likely to say they *remembered* an event they recalled vs. just *knowing* it occurred—a defining feature of autonoetic recall.^29^ Further, older children also gave more contextualized descriptions of their recalled memories and needed fewer cues to elicit recall. Thus, autonoetic recall may develop particularly late, undergoing significant development during the school years. In agreement, studies assessing source recognition in children (i.e., explaining how one knows something) also show significant development between the ages of 3 and 6.^4^^,^^94^

A further distinction may be made between strategic vs. spontaneous recall. Strategic recall tests simply involve asking what a child remembers about an event (Figure 5C). These tests place additional cognitive demands on the child, as they need to conduct a cognitive search for the correct memory. In other words, strategic recall requires mature executive function as well as mature memory trace recall. Spontaneous recall, on the other hand, is less cognitively demanding and is simply thought to arise via an associative process (Figure 5B)—e.g., walking past a park visited before may spontaneously elicit recall of one’s last visit to the park.

Interestingly, recent research suggests that spontaneous recall may develop early.^95^^,^^96^ Leichtman^96^ had children aged between 4 months and 3 years engage in a play event. The children returned to the lab 3–6 months after the learning episode. Leichtman found that children as young at 16–17 months of age spontaneously recalled their previous visit to the lab. Further, a series of subsequent experiments by Krøjgaard and colleagues^97^^,^^98^^,^^99^ showed that spontaneous recall is also present for events experienced only once. In their studies, Krøjgaard et al. had children aged 3–4 years engage in a play event. On a subsequent visit, occurring at least 1 week later, spontaneous retrievals of the encoding event were recorded by an experimenter. Importantly, on the second visit the children were not shown the toys they had played with on their first visit but were simply placed back into the same room. Krøjgaard et al. noted that 30%–60% of children displayed spontaneous recall for their first visit, and no age-related differences were observed. Importantly, when explicitly asked to recall their visit, a striking distinction in performance was observed between the younger and older children with the older children outperforming younger children.

Taken together, these studies suggest that spontaneous recall may mature before strategic recall. Specifically, spontaneous recall may be mature by age 3 at least, when strategic recall is still relatively immature. Thus, perhaps the late emergence of recall observed in multiple studies (e.g., Hayne and Imuta,^76^ Mastrogiuseppe et al.,^77^ and Scarf et al.^89^) could be due to the fact that these studies required strategic recall.

Differentiating between different forms of retrieval is not straightforward in non-human animals. However, spontaneous recall has been documented extensively in apes (e.g., Lewis et al.^100^). For example, when orangutans are placed back into an environment where they had found tasty treats hidden 2 weeks earlier and are presented with a distinctive cue of the learning event (food crumbs) they immediately searched for the food.^100^ Thus, perhaps animal researchers can differentiate between memory retrieval processes by assessing if memory requires an execution of a goal-directed behavior, which may reflect spontaneous recall processes, or whether it need only be expressed via novelty-related behaviors, which may reflect the engagement of recognition processes. Similarly, non-human animal researchers may draw on ingenious receiver-operating characteristic (ROC) analyses that are thought to distinguish between these different retrieval processes.^101^

## Development of the neural substrates and mechanisms for episodic memory

Here, we will provide a brief overview of a selection of studies that have charted the morphological and physiological development of the neuronal substrates implicated in episodic memory. As the hippocampus is known to be critical for episodic(-like) memory processes (e.g., Scoville and Milner^19^), this discussion will focus on its structural and functional development. A number of other brain regions have also been implicated in episodic memory development, such as the prefrontal,^45^ parietal,^102^ and parahippocampal cortices.^103^ Due to space constraints, however, we will not review their development here, but we refer the reader to Box 3 that describes the potential links between prefrontal and hippocampal/episodic memory development and point the reader to a number of excellent articles that have discussed the development of diverse neural circuits implicated in episodic memory.^17^^,^^32^^,^^45^^,^^102^^,^^104^^,^^105^ For more detailed reviews on cellular and molecular development of the hippocampus, we refer the reader to recent comprehensive reviews on the topic.^32^^,^^33^^,^^34^^,^^36^^,^^48^

### Neural substrates of episodic-like memory ontogenesis

In human adults, episodic memory is known to depend critically on the hippocampal formation.^19^ Seminal research has shown that injury incurred to the hippocampus early in life causes specific deficits to W-W-W memory function.^10^^,^^18^^,^^62^ Vargha-Khadem and colleagues showed that hypoxic-ischæmiasuffered in the peri/neo-natal period, that results in selective hippocampal atrophy,^10^^,^^106^^,^^107^ leads to a selective cognitive impairment to episodic memory (“developmental amnesia” [DA]). These individuals struggle to recall everyday events and special occasions, while general cognitive ability (language skills, factual knowledge, etc.) is relatively preserved.^108^ The memory capabilities of DA patients have been extensively studied. These studies have shown that DA patients can form simple item (what) memories, and even some forms of what-where spatial memories,^109^^,^^110^ although they seem to have a specific deficit in what-where encoding that requires an allocentric representation^111^ as well as a deficit in temporal encoding.^10^ Furthermore, their deficit in memory retention seems to be mediated via an inability to retrieve memories via (autonoetic) recall. If memory retention is assessed via recognition memory tests (e.g., multiple choice), DA patients often perform comparably to controls^112^^,^^113^ (see Box 2 for a further discussion of DA and its implication for developmental neuro-plasticity). Thus, the hippocampus seems critical for the development of allocentric what-where and W-W-W encoding, as well as recall, while the development of what and egocentric what-where memories, particularly when accessed via recognition processes, may be less dependent on the hippocampus.

Box 2 **Functional reorganization in the presence of early hippocampal injury**

Developmental amnesia (DA) is a limited form of amnesia resulting from selective, bilateral hippocampal atrophy associated with an early-life hypoxic-ischæmic episode.^10^^,^^106^^,^^107^^,^^114^ Individuals with DA reach age-appropriate developmental milestones across infancy to adolescence for language acquisition, semantic knowledge, motor, and educational achievements. However, a profound and chronic delay-dependent deficit in the ability to remember personally experienced events—including birthday parties, special occasions—usually becomes apparent around or shortly after the preschool years. The memory failures that become apparent are well illustrated by the following anecdote, related to one of the authors (FVK) by the mother of patient Jon—a DA patient whose amnesia has been extensively described.^10^

One day, when he was 8 years old, Jon went to the mall with his mother where he saw a woman wearing a sari with a large snake wrapped around her shoulders and arms. Jon was fascinated by the live snake. Noting his interest, the woman asked Jon to approach so he could touch and stroke the snake. Moments later, Jon and his mother left the mall and drove home. Upon entering the house, Jon’s mother asked him to “go tell your father what you just saw in the mall.” To which Jon responded with a puzzled look on his face: “what did I see?”

One of the most striking aspects of DA is that in the presence of this profound deficit in episodic recall (via autonoesis), the ability to learn facts and world knowledge remains intact and continues to develop with age and cognitive ability. In other words, DA is characterized by a striking dissociation between episodic and semantic memory.^10^^,^^108^ This dissociation is consistent with influential models of the organization of cognitive memory that posits the two memory systems are dissociable both functionally and neuroanatomically.^115^ Indeed, if a DA patient is asked about events of their daily life, they seldom respond with “I don’t remember.” Rather, they may give a generic account of events that are plausible to occur,^116^ suggesting memory encoding and consolidation is at least to some extent preserved in DA.

DA is a prime example of the ability of the developing brain to reorganize in response to injury. A seminal study by Maguire and colleagues^117^ showed that although the DA patient Jon displays, in adulthood, activation of the same network of brain regions on the left as in controls in relation to autobiographical memory retrieval,^118^ he additionally activates many homologous regions on the right, including the hippocampus, surrounding parahippocampal regions, and associated cortices. This suggests that in contrast to individuals with typical neurodevelopment, the autobiographical memory network in DA is bilaterally subserved. Furthermore, in controls autobiographical event memory retrieval was associated with increased functional connectivity between the parahippocampal cortex and hippocampus, whereas in Jon retrieval of autobiographical events displayed enhanced interaction between retrosplenial-hippocampal and retrosplenial-frontal cortices.^117^ Thus, while autobiographical event memory retrieval in DA activates the same brain regions as controls, the pattern of functional connectivity between the hippocampus and the cortex is different.

Recent evidence suggests that the extent of hippocampal damage in DA has a strong influence on the (re-)organization of the medial temporal lobe memory circuit. Specifically, the uncus (the most anterior part of the hippocampus) was found to be relatively preserved as compared with the other regions of the hippocampus,^106^ and the degree of sparing predicted memory impairments. Greater preservation of the uncus predicted worse recall (see below figure). These negative correlations suggest that when hippocampal circuits are only partially damaged (uncus preserved), the information flow may persist in the spared hippocampal circuits and results in incomplete, and possibly disruptive information processing.^106^ Together with the absence of structural damage in surrounding cortices, these negative correlations could reveal the existence of multiple, redundant information processing routes within the spared hippocampal/cortical areas. These parallel circuits could compete for control and disrupt memory performance. In contrast, greater hippocampal damage might induce greater compensatory reconfiguration and enable other structures to assume important aspects of memory function. Importantly, the compensation is not enough to rescue episodic memory function but rather may allow semantic memory to mature without the hippocampus, as has been found in DA patients.^10^

Putative compensatory mechanisms of the immature brain that rescue, and possibly augment,^109^^,^^119^ non-hippocampal-dependent mnemonic processes need to be investigated. Some initial insights may be gained from a recent study in NHPs, which suggest that cellular plasticity might occur in the parahippocampal gyrus following early hippocampal damage.^120^ The population of immature neurons present in layer III of the entorhinal cortex and layer II of the perirhinal cortex have been shown to increase in number in monkeys with neonatal bilateral hippocampal lesions, while the number of mature neurons was shown to increase in layer III of the entorhinal cortex. Perhaps this increase in neuronal number in the entorhinal cortices (and elsewhere) promotes the contribution of non-hippocampal memory processes and spares some aspects of memory function. We hope future research, which make use of non-human animal models of DA, will give further insight into the compensatory processes that occur in the presence of early-life brain injury.

**Figure:**
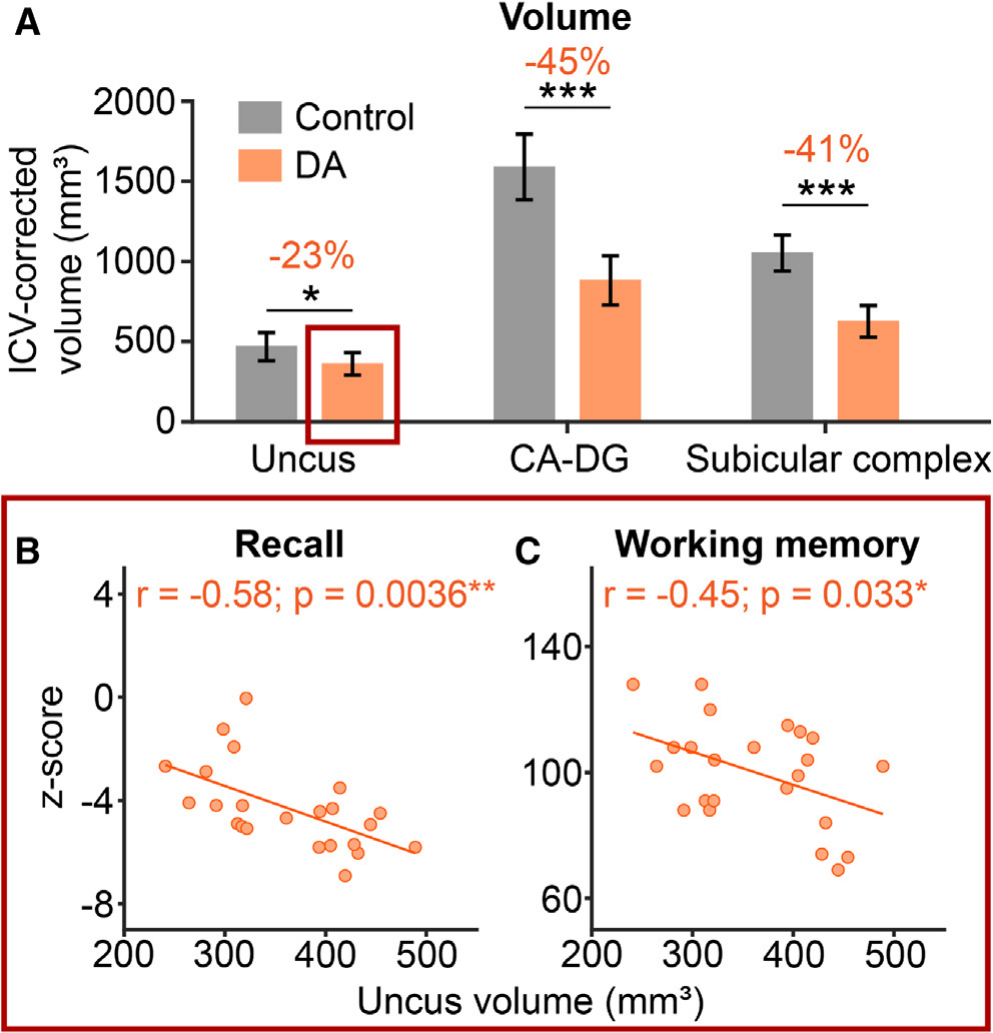
Volume of hippocampal regions and anatomo-functional correlations in DA (A) Volume of hippocampal subregions. Subregional differences between DA patients and controls compared with a twoway ANOVA. p values for pairwise comparisons corrected with a Tukey HSD post-hoc test. Error bars show standard deviation of the mean. ∗p < 0.05, ∗∗∗p < 0.001. (B) Pearson correlation between recall/working memory scores and uncus volume. Modified with permission from Chareyron et al.^178^

These results largely agree with lesion studies carried out on rodents and NHPs. Neonatal lesions to the NHP hippocampus have been found to impair the ontogenetic emergence of allocentric what-where memories, such as those tested via the object-in-place VPC task.^62^ Neonatal hippocampal lesions do not impair object (what) memory VPC learning, when this is tested within the first post-natal year. However, when tested at 18 months of age, these neonatally hippocampal-lesioned NHPs, as well as those whose hippocampi were lesioned in adulthood, show marked impairments relative to sham-operated monkeys.^62^ This fact suggests that the neuronal substrates supporting this building block of episodic-like memory undergoes significant reorganization during the first post-natal year in NHPs. Early in life, OR may be supported by perirhinal and parahippocampal cortices,^20^^,^^121^ allowing infant NHPs to encode the what in W-W-W associative memory despite the absence of the hippocampus, whereas later in life such memory requires the hippocampus.

Rodent lesion studies have also shown that the emergence of associative what-where spatial memory, as measured by the ability to learn a spatial alternation rule on the T-maze^18^ or to recognize a spatially displaced object,^42^ depends on the hippocampus early in life. Lesioning the hippocampus in the neonatal period has been found to abolish the development of these forms of what-where memory.^18^^,^^42^ Similar findings have been observed for what-where contextual memory development.^122^^,^^123^

In addition to the hippocampus, episodic-like memory development may also involve the prefrontal cortex (PFC). Neonatal hippocampal lesions have been found to influence the structural development of the PFC in NHPs and rodents.^42^^,^^124^^,^^125^ In Box 3, we elaborate further on the potential role of the PFC in memory development.

Box 3 **Prefrontal cortex and memory development**

Maturation of memory processes results in increased localization of function. Although the hippocampus is critical for memory development, the prefrontal cortex (PFC) is also strongly implicated. The PFC is thought to be critical for mature working memory capabilities as well as adult-like, strategic recall.^126^^,^^127^^,^^128^ It is therefore crucial to consider the PFC and its interactions with the hippocampus when tracing the emergent memory processes from infancy to their full maturation.

The PFC occupies the anterior pole of the mammalian brain and is involved in several cognitive processes, including working memory (WM) and rapid adaptation to various situations.^129^^,^^130^^,^^131^ Age-related improvements in WM have been documented from childhood to adolescence in humans, monkeys, and rodents^105^^,^^132^^,^^133^^,^^134^^,^^135^^,^^136^^,^^137^ and are believed to result from important anatomical, chemical, and functional changes occurring in the PFC from infancy to adolescence.^138^^,^^139^^,^^140^^,^^141^^,^^142^^,^^143^^,^^144^ Early in the post-natal period, overproduction of synaptic contacts and wiring occur, and synaptic density peaks relatively late (2–4 years of age in humans, 1–2 years in monkeys, and after the fourth post-natal week in rodents)^142^^,^^143^^,^^145^^,^^146^ and is then refined as development advances. Similarly, neuroimaging studies have indicated that gray matter (GM) volume increases across the cortex prior to puberty, reaching a peak around early to mid-pubertal period, followed by a post-pubertal decline during adolescence due to synaptic pruning.^147^^,^^148^ Despite a lack of precise homologies between specific PFC areas in primates and rodents,^149^ early investigation and genetic interventions in rodents have provided a deeper understanding of the PFC circuit wiring and its relationship to cognitive maturation (see Chini and Hanganu-Opatz^45^ for a review). Briefly, while the pruning of synaptic branches^150^ is modulated by molecular factors, electrical activity mainly controlled the refinement of connectivity with transient bouts of beta-low-gamma rhythmic oscillations generated by pyramidal neurons in the PFC supragranular layers.^151^^,^^152^^,^^153^ These bouts of electrical activity occur naturally in response to incoming stimuli from the hippocampus^154^^,^^155^ and appear to have important functional correlates.^151^^,^^154^^,^^156^ In addition, modulation of excitatory and inhibitory neurons within the PFC supragranular layers leads to further development of intrinsic PFC circuitry. Indeed, excitation/inhibition (E/I) imbalance during early adolescence results in severe cognitive deficits.^152^

Importantly, evidence indicates that many memory processes involve the participation of hippocampal-PFC interactions.^128^^,^^157^^,^^158^ During spatial working memory, hippocampal synchronous activity has been shown to slightly precede the activity of PFC neurons in humans, monkeys, and rodents.^159^^,^^160^^,^^161^^,^^162^^,^^163^ During the early stages of post-natal development, the functional maturation of the PFC is driven by other cortical and subcortical regions, including the patterns of coordinated activity generated by the hippocampus.^164^ Further, together with deficits in associative memory, early hippocampal insult yields WM impairment, which are not observed after hippocampal insult incurred in adulthood (rodents,^137^^,^^144^ monkeys,^165^^,^^166^ and humans^167^). Concurrently, these WM deficits are associated with long range neural changes in the PFC, such as reduced number of interneurons and decreased spine density of pyramidal prefrontal neurons,^168^^,^^169^ altered PFC firing patterns,^144^^,^^170^ decreased functional connectivity within the PFC cortical networks^171^ as well as anatomical connectivity between the hippocampus and PFC.^124^^,^^125^ Given the critical role of hippocampal rhythmic activity in the early post-natal period for normal PFC maturation, it is tempting to suggest that as a result of the early hippocampal lesions, plastic changes within the PFC may have resulted from a lack of hippocampal inputs during the post-natal period. Although the exact timing for the emergence of these plastic changes is still unknown, the above results stress the importance to design future studies on the critical cross-talks between the hippocampus and PFC during development to provide a more precise neural account for the emergent memory processes from infancy through late adolescence.

### Structural development of the neuronal substrates for episodic-like memory

**Figure 6:**
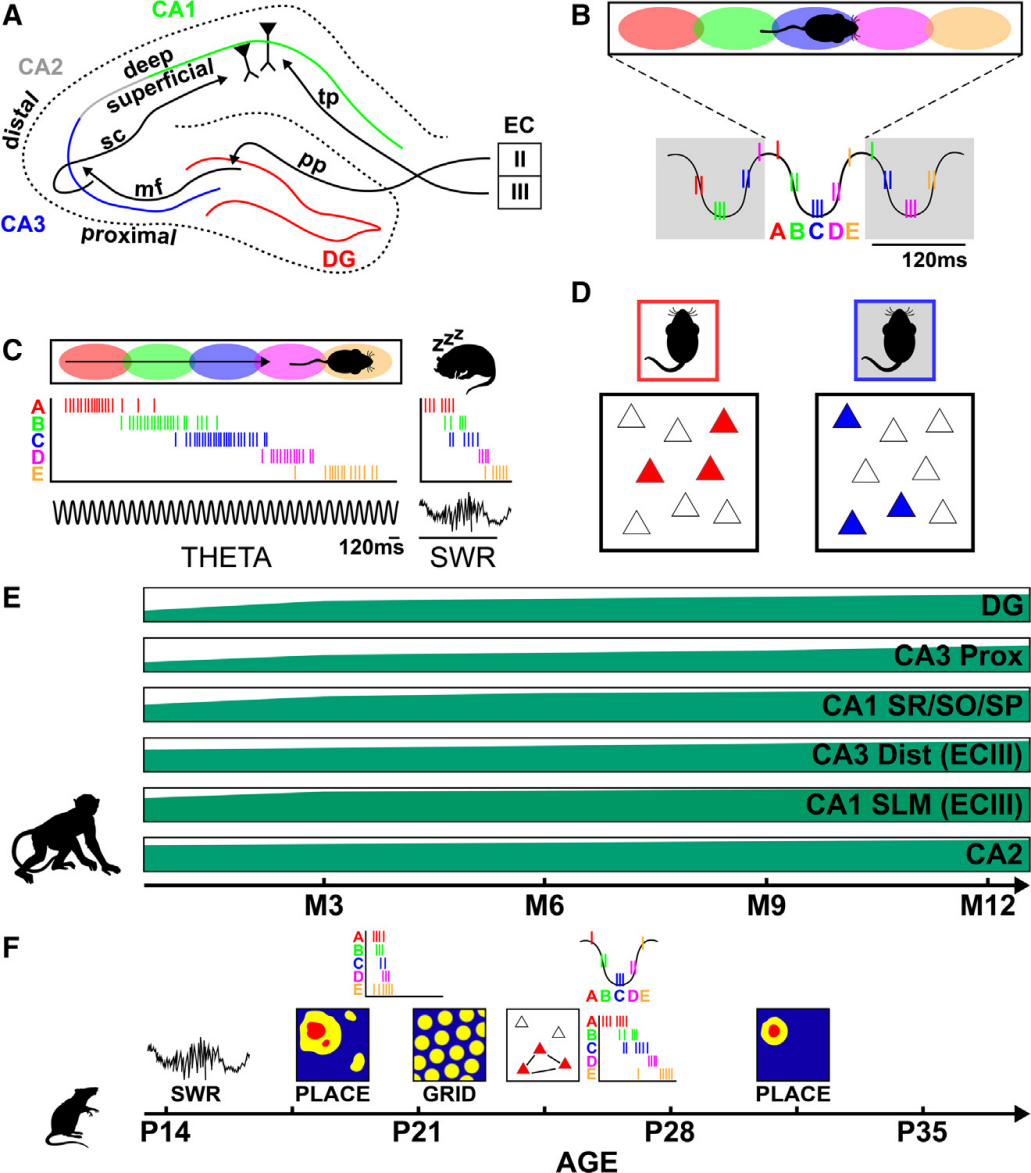
Development of hippocampal morphology and neurophysiological mechanisms (A) Anatomical diagram of the rodent hippocampus, its subfields, and principal intra and extra-hippocampal projections. (B) Schematic of hippocampal theta sequences. Top: colored circles represent place fields of individual place cells. Bottom: spikes from place cells are shown as rasters nested in theta oscillations. (C) Schematic of hippocampal replay. Left: runs on a linear runway are underpinned by sequential activation of place cells (top: schematic of place fields on track, bottom: raster plot of place cell activity during a single-track traversal). Right: during rest/sleep, place cell sequences observed during wakefulness are spontaneously replayed during sharp-wave ripple events. (D) Schematic of engram tagging. Top: rodent located in two environments. Bottom: colored triangles depict engrams (i.e., active cells) for the two environments. (E) Morphological maturation of the hippocampal CA and DG fields based on NHP volumetric analysis.^17^ Anatomical development of the rodent and human hippocampal CA and DG fields follow a similar order. (F) Developmental timeline of neurophysiological mechanisms supporting memory in rodents. CA1–3, cornu ammonis 1–3; DG, dentate gyrus; EC, entorhinal cortex; mf, mossy fiber pathway; sc, Schaffer collaterals; tp, temporoammonic pathway; pp, perforant pathway.

Converging evidence, obtained from different species, point to a protracted structural development of the hippocampus. The volume of the hippocampus is known to double in the first 2 years of human life^172^^,^^173^ with continued growth in the subsequent years,^174^ and possibly into early adolescence.^172^ However, the different subfields—cornu ammonis fields 1–3 (CA1–3) and dentate gyrus (DG) (Figure 6A)—of the hippocampus are thought to develop at different rates, with area CA2 likely being relatively mature at birth, while the CA1 and CA3 fields and DG develop significantly in the post-natal period.^174^ The development of DG is known to be particularly protracted, lasting into the 2nd post-natal year at least.^175^^,^^176^ Recent volumetric measurements of the DG and CA fields indicate that CA3 and DG may continue to develop in size until early adulthood.^177^

Stereological studies on developing NHPs and rodents have revealed similar sub-field developmental gradients. The volume of the NHP CA1 has been found to be relatively mature by 6 months of age while DG and CA3 continue to develop until the 2nd post-natal year, at least.^103^^,^^179^ Up to 40% of hippocampal DG neurons are thought to be born postnatally in NHPs^103^^,^^179^ (but see Raki^180^). Similarly, peaks in neurogenesis occur in the post-natal period in rodents.^181^ Donato and colleagues charted the molecular maturation—assessed by analyzing expression of doublecortin, parvalbumin (PV), and synaptic puncta—of hippocampal and entorhinal cells and found the DG was the last to develop of all hippocampal subregions.^182^ Research in non-human animals has also highlighted developmental changes within subregions. For example, the volume of distal CA3 (closer to CA2) becomes adult-like before proximal CA3 (closer to DG), with the volume of distal CA3 being relatively mature at birth while the proximal CA3 continues to mature beyond the first post-natal year in NHPs.^103^ Similar results have been observed in rodents. Distal CA3 neurons are born earlier than proximal CA3 neurons and enter into the hippocampal circuit at an earlier developmental stage.^183^ Of note, distal CA3 receives direct innervation from the EC, whereas proximal CA3 is more extensively innervated by the later maturing DG.^184^

Within CA1, NHP stereological studies have shown the sublayer stratum lacunosum-moleculare (SLM), receiving direct projections from layer three of the entorhinal cortex (ECIII), matures before the other sub-layers (stratum radiatum/pyramidale/oriens [SR/SP/SO]), which are preferentially targeted by CA3 afferents^103^ as part of the tri-synaptic loop. Specifically, SLM reaches adult-like volume by 3 months of age in NHPs, whereas the other layers develop through the first post-natal year. Rodent studies have observed a deep-to-superficial gradient for neurogenesis and migration in CA1. Deep cells are born and migrate earlier than superficial neurons.^183^ Although the cell bodies of the later maturing superficial CA1 cells are located closer to the early maturing SLM sublayer of CA1, electrophysiological studies in adult rodents have shown that these cells are preferentially targeted by the late maturing CA3 fibers.^185^

Together, these results suggest that the maturation of the hippocampal CA fields may be shaped by the maturation of their dominant inputs with subregions receiving direct projections from ECIII (SLM of CA1 and distal CA3) maturing before areas that are more heavily innervated via the tri-synaptic pathway (DG, SR/SP/SO of CA1 and proximal CA3). See Figure 6E for a timeline of the morphological development of the NHP hippocampus. In terms of the relation between these structural developmental gradients and W-W-W memory development, perhaps the early maturing (sub)regions support rudimentary episodic-like memory function—such as spatial what-where memory using an egocentric reference frame and imprecise contextual what-where memories. As the DG may be the last to reach maturity, one may speculate that the late development of the full W-W-W encoding triad may depend on this late maturing region. In Box 4, we speculate further on the possible links between structural development of the hippocampus and W-W-W memory maturation.

### Development of the neurophysiological mechanisms for episodic(-like) memory

In recent years, significant methodological advances have been achieved that now allow researchers to image the developing human brain. These studies have highlighted that the hippocampus may support episodic-like memory early in the post-natal period. Prabhakar and colleagues^187^ imaged brain activity via functional magnetic resonance imaging (fMRI) in 2-year-old toddlers during sleep. During scanning, sounds associated with a previous encoding session (playing with a soft toy) were played. Prabhakar and colleagues observed increased hippocampal activity in response to the presentation of the learned song vs. a novel song.^187^ Further, the amount of hippocampal activity correlated with the children’s recall accuracy of the toy-sound association (see also Mooney et al.^188^). Interestingly, hippocampal activity was also observed when testing occurred several months later, despite the children not being able to recall the toy-sound association.^189^ Ellis and colleagues found the hippocampus to be engaged while infants as young as 3 months old observed temporally ordered visual stimuli.^173^ However, they did not test memory.

A dissociation between hippocampal activity and the ability to recall what-where memory has also been noted in DA.^110^ Elward and colleagues had DA patients learn word-scene associations and then tested them on their what-where memory (via a recall test) before a fMRI scanning session. The authors found that, despite the DA patients displaying poor recall, the presentation of the paired scene elicited hippocampal activity similar to controls. Overall, it appears likely that, although the human hippocampus undergoes significant maturation in the early post-natal period, it may still support elemental W-W-W memory function during this period, even though the behavioral expression of memory is still absent.

Neurophysiological research in developing rodents, carried out over the past decade, has started to elucidate the ontogeny of hippocampal neuronal mechanisms, which may give insight into this early involvement of the hippocampus in W-W-W memory. Below, we summarize core findings from this research. Electrophysiological studies in rat neonates (post-natal weeks 1–2) have shown that major hippocampal oscillations, namely, theta (6–12 Hz) and gamma (30–100 Hz) are already present by 2 weeks of age, yet they continue to increase in frequency and power during the third and fourth post-natal week.^22^^,^^154^^,^^190^^,^^191^ Sharp-wave ripple (SWR) oscillations display a more protracted emergence. The sharp-wave component—originating from synchronous activity in CA3—may emerge in the first post-natal week^192^^,^^193^ whereas the high frequency ripple component—reflecting population activity bursts in CA1—has only first been recorded at ∼2 weeks of age and continues to develop in power until the end of the third post-natal week.^27^ Thus, by 3–4 weeks of age, major hippocampal oscillations that orchestrate information processing in hippocampal circuits seem to have matured to near adult-like levels.

Furthermore, several studies have charted the development of hippocampal spatially tuned neurons (“place cells”) in rat pups. Principal cells of the CA regions of the adult hippocampus display spatially confined activity during locomotory periods encoding allocentric relations between an animal’s current location and landmarks/environmental boundaries.^194^^,^^195^ Cells with similar functional tuning have been observed in humans and NHPs, albeit in smaller numbers and often displaying a conjunctive activity correlate—e.g., coupling to location as well as other spatial variables (head direction, spatial view, and velocity).^196^^,^^197^^,^^198^ Hippocampal place cells can be recorded, in the rat, from P16. However, their stability as well spatial specificity continues to mature until at least the end of the fourth post-natal week.^22^^,^^23^^,^^199^^,^^200^ Interestingly, around the time grid cells from ECII emerge (∼P20)^22^^,^^23^ (which provide place cells with spatial input), place cells start to form more stable spatial firing fields in the center of open-field environments. Prior to this age, place cells are only stable around environmental edges. Furthermore, Muessig and colleagues showed that although place cells in the pre-weaning (<P21) period remap between different environments (alter where they fire), remapping seems to be driven by olfactory cues at this age, and only in the fourth post-natal week do place cells start to integrate multiple sensory cues.^201^ It should be noted, however, that integration of more subtle sensory cues (such as floor texture) is still not apparent in the early post-weaning period (beginning of 4th post-natal week).^201^ Thus, although place cells are present during the third post-natal week, their stability and ability to integrate multi-sensory cues extends at least until the end of the fourth post-natal week.

The development of hippocampal “replay” (Figure 6C)—time-compressed reactivations of CA1 wakeful activity patterns thought to support the commission of new memories to long-term storage^202^^,^^203^^,^^204^^,^^205^—has recently received increased attention. Reactivations have been observed as early as P17. However, early reactivations tend to depict confined regions of an animal’s environment.^25^^,^^26^ It is only during the fourth post-natal week that replays start to tie together sequentially visited locations,^25^^,^^26^ akin to what is observed in adult rats. In tandem with the emergence of adult-like, sequential replay, place cells start to show sequentially organized activity within individual cycles of theta-band oscillations^25^ (“theta sequences”; Figure 6B). Overall, by ∼fourth post-natal week the neural machinery thought to support memory encoding and retention seems to be in place.

Significant progress has also been made to tracking encoded memory “engrams”^206^ during the early post-natal period. These studies capitalize on techniques that allow tagging of neurons active during encoding periods^207^ (Figure 6D). Using this technique, Guskjolen and colleagues reported that although contextual associative memories (tested via CFC) encoded at P17 are rapidly forgotten in natural settings, if the original engram is optogenetically reactivated in adulthood, mice display intact recall of the fearful early-life event.^57^ Similar results were obtained by Power and colleagues,^87^ who replicated the effect for OR and a cued version of the Barnes maze (where rodents learn the location of an escape hole on a circular platform). Subsequently, Ramsaran and colleagues^24^^,^^92^ have shown that infantile memories are less precise and engrams include more CA1 neurons than in adults.^24^ The reduction in CA1 engram size observed in development may reflect the protracted development of inhibition in the hippocampus. Indeed, Ramsaran et al. only observed adult-like inhibition, mediated by PV expressing interneurons, during fourth post-natal week and artificially decreasing PV activity in adult animals was found to lead to pup-like, enlarged engrams and poor what-where memory. In sum, by the middle of the fourth post-natal week, memory engrams seem to have matured.

Together, current work suggests that by ∼2 weeks of age rudimentary rodent hippocampal function is present, as all major oscillations are present and place cells can be recorded. However, during the subsequent ∼2 weeks, rodent hippocampal representations and network mechanisms undergo significant development, with the emergence of adult-like spatial tuning, sparse engrams, and sequential activity patterns (see Figure 6F for a summary developmental timeline). In terms of how these neurophysiological changes relate to the gradual developmental emergence of W-W-W memory, it may be that basic place cell function, stationary replay and dense memory engrams may support elementary W-W-W memory—such as egocentric/imprecise what-where memories. The maturation of allocentric and precise what-where memories as well as complete spatial-temporal (W-W-W) memory encoding coupled with the ability to retain memories over extended periods of time may depend on a more precise hippocampal neuronal code and mature replay (see Box 4 for further discussion on links between neurophysiological and W-W-W memory development).

Box 4 **Linking neuro- and episodic-like memory development**

Links between neuro- and cognitive development remain relatively unclear. Drawing on the extensive literature described here, we will speculate on these links. We emphasize that the links we suggest are hypothetical and need to be tested experimentally.

As object (what) encoding is known to develop first in mammalian W-W-W memory development, we speculate that this function may be supported by the early maturing subregions of the hippocampus, such as CA2, deep CA1, and distal CA3 as well as the perirhinal cortex, which has been shown to be critical for object encoding early in life.^20^ As no *in vivo* neural recording study has targeted these specific regions, the neuronal representations that underlie the early emergence of this fundamental component of W-W-W memory remain to be elucidated.

In terms of what-where (spatial and contextual) encoding, we hypothesize that the emergence of this ability depends on the maturation of place cells and the full tri-synaptic loop (i.e., ECII → DG → CA3 → CA1). Rudimentary what-where encoding, such as the ability to discriminate between different environments, may be possible before place cell representations become stable and are able to integrate multi-sensory cues and when hippocampal engrams are dense, such as has been observed during the third post-natal week in rodents.^22^^,^^23^^,^^24^ However, we conjecture that mature what-where encoding, where animals can for example disambiguate environments whose sensory features overlap, depends on the maturity of the tri-synaptic loop and in particular the DG which may lead to a precise spatial CA1 code and a sparse engram, which in the rodent is observed from the fourth post-natal week onward.^22^^,^^23^^,^^24^

Temporal (what-when) encoding in W-W-W memory, we speculate, may depend on the emergence of sequential encoding in CA1 (theta sequences and replay). Such sequential neuronal encoding has been found to develop relatively late, with the earliest inflection point found in fourth post-natal week in rodents.^25^ This agrees largely with the human literature on what-when encoding, which has also displayed a late inflection point (e.g., Hayne and Imuta^76^ and Scarf et al.^89^).

In terms of the neurobiological basis of memory retention, we speculate that this ability depends on the development of hippocampal reactivations and replay (which in turn depend on the maturation of SWRs). Reactivation of single locations have been reported in the third post-natal week in rodents.^25^ However, we reason that due to the immaturity of CA1 representations at this developmental stage, reactivations are not able to support long-term associative memory retention when they first emerge. Only when place cells start forming stable, specific, and sequential functional representations do reactivations start supporting the retention of W-W-W memories. Further, we hypothesize that the development of memory retention may also depend on the maturation of hippocampal projections to the cortex. Hippocampal-cortical communication during reactivations is thought to play a critical role in the stabilization of new memories (e.g., Maingret et al.^186^). Studies have shown that the deep layers of the entorhinal cortex (ECV/VI)—the primary hippocampal-cortical output region—develop late.^103^ As such, it may be that adult-like long-term retention only emerges once this extra-hippocampal communication pathway has developed.

## Challenges to comparative research

We have described the ontogeny of key cognitive and neurobiological processes that contribute to the gradual development of episodic-like memory. However, several methodological and theoretical issues make it hard to determine developmental trajectories with precision. Here, we summarize these challenges and offer potential solutions.

### Comparative potential of (memory) tasks

Many neurophysiological studies in rodents have not investigated neuronal maturation in the context of behavioral data on memory development (rodents simply shuttle back and forth on linear runways or forage for food^22^^,^^23^^,^^25^^,^^199^). Other studies use tasks that are not implementable in developing humans, such as CFC. Much behavioral research on children has used paradigms such as the mobile conjugate reinforcement task^50^ and deferred imitation,^8^ which draw on a motor and social repertoire not available to other species. We encourage researchers to use tasks (e.g., VPC, object exploration tasks) that are amenable to cross-species testing so that insights gained from one species can be more readily transferred to other species.

### Episodic nature of memory tests

Further, when memory tests are employed in developmental research, it is critical that animals are not over-familiarized with study stimuli, to ensure that memory for unique events (i.e., "one-shot” learning^208^) is indeed being tested and to use stimuli configurations/sequences that are not already familiar to the developing animal. For example, encoding sessions should be limited in duration, as is routinely done in CFC and OR studies (e.g. OCR, OPC, OPCR, and VPC) (e.g., Campbell and Campbell,^55^ Guskjolen et al.,^57^ Ramsaran et al.,^59^ and Asiminas et al.^60^). However, extensive training is also common—for example, in watermaze studies,^69^^,^^70^ even though one-session paradigms exist for adults^209^ and could be optimized for use in developing animals. Failure to limit encoding sessions may obscure the specific type of memory being assessed.

Relatedly, although CFC tasks are often used as a proxy for episodic memory, they are also known to engage different neural circuits than non-affective episodic-like memory tasks.^210^^,^^211^ This fact raises questions about whether this task specifically tracks episodic memory ontogeny. We urge memory development researchers to not exclusively rely on CFC paradigms when studying W-W-W memory development and if using CFC to also run parallel studies using non-affective W-W-W memory tasks to ensure insights gained transfer to other types of episodic-like memory and to enhance cross-species impact.

### Measuring encoding success

The success of encoding—assessed via memory testing immediately after an encoding session—is not consistently determined (e.g., memory is sometimes exclusively tested after a delay, e.g., Ramsaran et al.^24^ and Mastrogiuseppe et al.^77^). Doing so complicates interpretations when memory fails at testing, as the failure could either be due to a retention or an encoding problem. Thus, we encourage memory development researchers to assess success of encoding immediately after a learning session. Alternatively, in some incidental learning paradigms (such as the VPC) encoding success can be directly gauged by measuring if (visual) exploration time reliably decreases (habituates) to study stimuli during encoding. As encoding and retention are known to develop at different time points, separating these component memory processes in experimental studies is critical to understand the ontogeny of episodic-like memory.

### Memory specificity

A related issue is the specificity of the encoded memory. For example, CFC studies have shown that early in life rodents tend to generalize fear responses to neutral contexts (e.g., Akers et al.^56^). Thus, it is essential to test the specificity of the contextual what-where association in CFC tasks. Similarly, tasks that are intended to measure spatial what-where encoding within an allocentric reference frame (e.g., Blue et al.^62^ and Guskjolen et al.^70^), can often be solved without the use of allocentric strategies.^67^^,^^69^^,^^121^ We encourage experimenters to use robust controls to disambiguate the nature of encoding such that the ontogenetic timeline of different facets of episodic-like memory function (e.g., generalization vs. specificity, egocentric vs. allocentric encoding) can be delineated. Importantly, tests of memory specificity need to take place both immediately after encoding as well as after a retention interval to ascertain if the development of distinct facets of episodic memory differ for different component memory processes (encoding vs. retention).

### Measuring retrieval processes

Retention of episodic-like memories is supported by different retrieval processes (recognition, spontaneous, and strategic recall). However, which retrieval process a memory task draws on is generally not tested in developmental studies. For example, when an animal is placed back into a study environment in OR studies, novelty preferences may be mediated either by recognition or recall processes. Similar arguments can be made for CFC studies and VPC studies carried out in humans and NHPs. We encourage researchers to develop tests that explicitly assess which retrieval process underlies memory performance so that the ontogeny of memory retention can be better understood. For example, by assessing memory via goal-directed behavior (e.g., an animal running to a safe region of an environment in CFC studies^91^) or using ROC curves.^101^ Delineating the developmental timeline of distinct retrieval processes may also help resolve contradictory findings regarding the age at which developmental inflection points for memory retention occur.

## Conclusions and future perspectives

Concerted research efforts over the past decades have significantly advanced our understanding of the ontogeny of W-W-W memory in different mammalian species and have provided insights into some of the key neurodevelopmental milestones that may support W-W-W memory ontogenesis. These studies have highlighted robust similarities between the ontogeny of W-W-W memory and its underlying neural circuits in different mammalian species. However, significant caveats remain. Particularly, we still lack information as to the development of different retrieval processes (recognition, strategic vs. spontaneous recall) and the different dimensions of episodic memory encoding (what-when and W-W-W). Consequently, the extent of overlap and variation in memory development processes across different mammalian groups remains to be fully ascertained. Further, the relationship between neuronal and memory development has only recently started to be elucidated (see Box 5 for further discussion on knowledge gaps). This last point is particularly pertinent as elucidating this relationship may have significant implications for understanding the neurobiological basis of common neurodevelopmental disorders affecting memory (Down syndrome, autism spectrum disorder, etc.) and impairments observed in the context of early-life brain injury (e.g., DA^10^).

Box 5 **Critical knowledge gaps**

**The development of memory retention retrieval process**: the ontogeny of different memory retention and retrieval processes (recognition, spontaneous/strategic recall) has not been studied systematically in any species. We encourage the non-human animal research community to develop tests that tap into different retrieval processes.

**Temporal (“when”) memory**: relatively little research attention has been devoted to this component of W-W-W memory, and much research thus far has focused on the sequential aspect of “when” encoding (the order in which events unfold in an episode). However, recalling when an episodic memory occurred also involves integrating incidental features (spatial or non-spatial) present at the time of encoding (e.g., floor color/texture, smells, emotions, etc.). Features that are collectively termed the spatiotemporal context.^212^ By definition, these do not include allocentric spatial cues or the location per se of where an event took place or relate explicitly to the event itself. We encourage researchers to pay closer attention to how such contextual encoding unfolds early in life.

**The development of core computational processes for memory**: how does the gradual development of W-W-W memory and adult-like retrieval mechanisms relate to key mnemonic computational processes such as pattern completion and separation?

**Relationship between neuro- and cognitive development**: we do not know how the emergence of mature neuronal function (e.g., place cells, replay) relates to the development of W-W-W/episodic-like memory capability. Further, the relationship between structural development and memory development is largely unexplored.

**The relationship between sleep and memory development**: the contribution of sleep to learning is known to undergo developmental changes.^53^ It is of critical relevance to study how sleep contributes to episodic memory ontogeny.

**Functional specialization in development**: a wealth of research has shown that the localization of function in the developing brain is not the same as in the adult. Relatedly, other brain regions—such as the PFC—are implicated in core aspects of W-W-W/episodic memory. The role extra-hippocampal regions play in memory development and changes to functional specialization have hitherto received limited research attention.

**Link between the development of executive function and episodic-like memory**: the emergence of mature W-W-W/episodic memory—and particularly strategic recall—may not simply depend on the maturation of mnemonic processes but also executive function. We encourage the research community to explore the role of general cognitive abilities in memory development.

Linking cognitive and neural development requires closer ties and collaboration between cognitive scientists studying human development and neuroscientists investigating neuronal circuit maturation in non-human animals. Specifically, we advocate for collaborative partnerships between human and non-human animal researchers where memory development can be studied in parallel in multiple species and tasks used to test memory capability are aligned as much as possible. Doing so would be a significant stepping-stone in establishing a comprehensive cognitive-neurobiological model of memory ontogeny and would ensure findings obtained via non-human animal research can be generalized to humans and ultimately benefiting society.
